# Inference of gene regulatory subnetworks from time course gene expression data

**DOI:** 10.1186/1471-2105-13-S9-S3

**Published:** 2012-06-11

**Authors:** Xi-Jun Liang, Zhonghang Xia, Li-Wei Zhang, Fang-Xiang Wu

**Affiliations:** 1 School of Mathematical Sciences, Dalian University of Technology, Dalian 116024, China; 2 Dept. of Mathematics and Computer Science, Western Kentucky University, Bowling Green, KY 42101, USA; 3 Department of Mechanical Engineering, University of Saskatchewan, 57 Campus Dr., Saskatoon, SK S7N 5A9, Canada

## Abstract

**Background:**

Identifying gene regulatory network (GRN) from time course gene expression data has attracted more and more attentions. Due to the computational complexity, most approaches for GRN reconstruction are limited on a small number of genes and low connectivity of the underlying networks. These approaches can only identify a single network for a given set of genes. However, for a large-scale gene network, there might exist multiple potential sub-networks, in which genes are only functionally related to others in the sub-networks.

**Results:**

We propose the network and community identification (NCI) method for identifying multiple subnetworks from gene expression data by incorporating community structure information into GRN inference. The proposed algorithm iteratively solves two optimization problems, and can promisingly be applied to large-scale GRNs. Furthermore, we present the efficient Block PCA method for searching communities in GRNs.

**Conclusions:**

The NCI method is effective in identifying multiple subnetworks in a large-scale GRN. With the splitting algorithm, the Block PCA method shows a promosing attempt for exploring communities in a large-scale GRN.

## Background

Rapid advances in high-throughput DNA microarray technology generate a huge amount of time course gene expression data which, in turn, calls for efficient computational models to characterize the network of genetic regulatory interactions. A number of methods have been proposed to infer GRNs from gene expression data. Boolean networks [1] use two states, "ON" or "OFF" to represent the state of each gene, and each state at the next time step is determined by Boolean logical rules. Bayesian Networks [2] infer causal relationships between two genes according to conditional probability functions. The stochastic nature makes them more accurate in modeling the dynamics and nonlinearity of gene regulation in large-scale systems. Bayesian Networks, however, usually do not include cycles and, thus, are difficult to deal with feedback motifs. Ordinary differential equations (ODEs) models [3-5] overcome this problem by modeling GRNs as a set of differential equations. Some other models such as signed directed graphs, multiple regression, state space model, etc., are addressed in the survey [6].

Whereas most of the existing work focuses on small-sized GRNs, limited attention has been given to interactions among large scale genes. Conventional approaches are usually designed for the network with connectivity less than a small fixed number [7]. Computational complexity is a major obstacle in reconstruction of large scale GRNs as determining the parameters in such a network is time-consuming. Sparsity is a common assumption used in modeling GRNs to reduce the computational complexity. Typically, in a sparse network, one gene interacts with only a couple of genes [7].

Recently, Yuan et al. [8] proposed a directed partial correlation (DPC) method for regulatory network inference on large-scale gene data. The DPC method combines the directed network inference approach and Granger causality concept for causal inference on time series data to reconstruct large-scale GRNs. Although modular discovery was provided by biclustering in gene expression data, the DPC method cannot present multiple sub-networks simultaneously.

We propose the NCI method for subnetwork identification by detecting community structures from large-scale gene expression data. Usually, GRNs have community structures: genes in the same groups are found with high density of "within-group" interactions and genes in different groups with low density of "between-group" interactions [9]. Many algorithms have been proposed to detect community structures by clustering [9-15]. To accomodate the large-scale GRN inference, we particularly propose a block principal component analysis (Block PCA) method, which explores community structure information for the NCI method.

The NCI method repeats two steps: (1) N-step: identify possible gene regulatory networks; (2) C-step: estimate community structure. At the N-step, a convex quadratic programming, formulated for the community structure, is solved to infer possible GRNs. This quadratic programming can be identically divided into *n *(the total number of genes) sub-problems, each of which has a much smaller dimension, and, thus, adapt to large-scale networks. At the C-step, the NCI method estimates community structure from the GRNs identified at the first step. When the algorithm terminates, a network with community structures is obtained.

## Methods

### An ODE model for GRNs

The processes of transcription and translation in a GRN consisting of *n *genes can be modeled as the following dynamic system:

(1) $$\begin{array}{c} \dot{x}=Cx+Sr\\ r=f(x), \end{array}$$

where vector *x *= [*x*_1_, *x*_2_, ..., *x_n_*]*^T ^*∈ ℝ*^n ^*is the concentration of mRNAs of *n *genes, *C *= *diag *[-*c*_1_,-*c*_2_, ..., - *c_n_*] ∈ *R^n × n^, c_i _*represents the degradation rate of gene *i, r *= [*r*_1_, *r*_2_, ..., *r_n_*]*^T ^*∈ ℝ^n ^is the reaction rates which is a function of concentrations of some mRNAs, and matrix *S *∈ *R^n × n ^*represents the stoichiometric matrix of the biological network. For simplicity, one can assume the reaction rate *r *is a linear combination of mRNAs concentrations. Let *F*∈ *R^n × n ^*be the coefficient matrix. Then,

(2) r=Fx.

By substituting (2) into (1), we have

(3) $$\dot{x}=Cx+SFx.$$

A standard discretization of system (3) by using the zero-order hold method on *m *observation points for a given sampling time Δ*t *is

(4) $$x\left(k+\text{1}\right)=Ax\left(k\right),$$

where *A *= *e*^*C*Δ*t *^+ (*e*^*C*Δ*t *^- *I*)*C*^-1^*SF*.

Let *X *∈ ℝ*^n × m ^*be a matrix of gene expression data, with the columns being the measured gene expression levels at *m *time points, and *n *being the number of genes. Let *X*_1 _and *X*_2 _be the sub-matrices of *X *made up by the first *m - *1 columns and last *m - *1 columns of *X*, respectively. According to [16], the gene regulatory network can be inferred by solving the following optimization problem:

(5) $$\begin{array}{cc} \min_{A} & {∥AX_{1}-X_{2}∥}_{2}^{2}\\ \text{s}\text{.t}\text{.} & A\;\text{is stable,} \end{array}$$

where ||·||_2 _is the Euclidean norm. Stability is usually used as a criterion to determine the qualification of the inferred GRN. For discrete models, *A *= (*a_ij_*)_*n × m *_is stable if

(6) $$\overset{n}{\underset{j=1}{\sum }}\;|a_{ij}|\;\leq \;1,\;\text{for all}\;i\;=\;1,\ldots ,n.$$

Moreover, since the network is commonly recognized as sparse, *l*_1 _regularization is added to Eq. (5) to obtain a sparse matrix *A*. Hence, with the sparsity and stability conditions, (5) becomes

(7) $$\begin{array}{c} \min_{A}\;\;{∥AX_{1}-X_{2}∥}_{2}^{2}+\gamma {∥A∥}_{1},\\ \text{s}\text{.t}.\;\;\;\sum_{j=1}^{n}\left|a_{ij}\right|\;\leq 1,\text{for all}\;i=1,\ldots ,n \end{array}$$

where γ is a positive scalar, ||*A*||_1 _= ∑_*i, j *_|*a_ij_*| is the *l*_1_-norm of matrix *A*.

### The NCI method

Since rows of *A *are independent in the objective function and constraints, problem (7) can be divided into *n *sub-problems and solved individually [16]. However, such a solution does not consider the information of community structure which implies multiple subnetworks. In this section, we propose the NCI method to overcome this problem. An observation is that interactions between genes in a community occur more frequently than those between different communities. We introduce a weighted matrix *W *= (*w_ij_*)_*n × m *_to distinguish genes in the communities with those outside. *w_ij _*is assigned a small positive value or zero if gene *i *and *j *located in the same community; a relatively large value, otherwise.

By adding term 〈*W*, |*A*|〉 to (7), we have

(8) $$\begin{array}{c} \min\;\;{∥AX_{1}-X_{2}∥}_{2}^{2}+\gamma {∥A∥}_{1}+\mu \langle W,|A|\rangle \\ \text{s.t.}\;\;\sum_{j=1}^{n}|a_{ij}|\;\leq 1,\text{for all}\;i=1,\ldots ,n, \end{array}$$

Where *μ *> 0 is a penalty parameter, |*A*| = (|*a_ij_*|)_*n × m*_, 〈*W*, |*A*|〉 = trace (*W^T ^*|*A*|) = ∑_*i, j *_*w*_*ij*_|*a_ij_*|. All elements of matrix *W *are nonnegative.

We propose a clustering method, named *Block PCA*, to update weight matrix *W*. With Block PCA, we can obtain matrix L*, reflecting the community structure of its corresponding network. Then, weight matrix *W *can be updated by

(9) $$W=\;1_{n,n}-L^{*},$$

Where $1_{n,n}\in {\mathbb{R}}^{n\times n}$ is the matrix with all 1's.

For example, consider a network with five nodes and

$$L^{*}=\left[\begin{array}{ccccc} 1 & 1 & 1 & 0 & 0\\ 1 & 1 & 1 & 0 & 0\\ 1 & 1 & 1 & 0 & 0\\ 0 & 0 & 0 & 1 & 1\\ 0 & 0 & 0 & 1 & 1 \end{array}\right].$$

Node 1, 2, and 3 form a community, and node 4 and 5 form another community. Particularly, we apply sparse singular value decomposition (SSVD) [17] on a general L* to identify the communities in GRNs. The NCI method is summarized in Algorithm 1.

**Algorithm 1:** 

Algorithm 1: The NCI algorithm	
**Input: ***X*;	
**Output:**	matrix *A *and communities of the GRN;
**Step 0**.	(Initiation) *W *: = 0 ∈ ℝ*^n × n^*. Select *μ *> 0, *γ_τ _>*0, λ_1_∈ (0, λ_2_).
**Step 1**.	(N-step: identify possible **N**etworks) Solve (8) to find an approximate matrix *A*.
**Step 2**.	(C-step: estimate **C**ommunity structure) Calculate weighted matrix *W*1 by Eq. (13), then solve the proposed block PCA model (12) to calculate L*.
**Step 3**.	(Update weight matrix) Update *W *by Eq. (9). If stop criteria are not satisfied, go to Step 1.
**Step 4**.	(GRN identification) Identify the communities of the computed GRN by SSVD.

Some additional details about Algorithm 1:

1. Stop criteria. The NCI algorithm stops when either of the following two criteria meet. (1) Weighted matrix *W *converges, that is, ||*W*^(*k*) ^- *W*^(*k*+1)^|| ≤ *tol *for a pre-defined constant *tol *> 0, where *W*^(*k*) ^denotes *W *at iteration *k*; (2) The number of iteration reaches the threshold.

2. The efficiency of the algorithm mainly depends on the estimation of the community structure of the underlying GRN. Since matrix *A *in (8) provides a base for estimation of community structure L* computed by (12), a poor estimation of *A *may result in an inaccurate *W*. Hence, instead of using only one estimation of *A*, we average out the errors by calculating a series of estimations with different arguments *γ *in (8) and combining them together. More specifically, we choose a set of *γ*_1_, ..., *γ_q _*and compute the corresponding solutions *A*^1^, ..., *A^q^*. Then, *A *in Step 1 of Algorithm 1 is set as

$$\begin{array}{ccc} a_{ij} & =A_{ij,}^{P} & \\ & \text{with}\;p=\arg\text{max}\left\{|A_{ij}^{u}||u=1,\ldots ,q\right\},\forall i,j. \end{array}$$

After the iteration terminates, model (8) is solved again to compute the matrix *A *with *γ *= *γ_τ_*, where *γ_τ_*, is a parameter in Algorithm 1.

3. The complexity of the subproblem is our primary concern about the design of the NCI algorithm. Since the subproblems may be called iteratively in Algorithm 1, the complexity of the NCI algorithm is determined by those subproblems. Both sub-problems (8) and the Block PCA model are convex, and can be efficiently solved by CVX [18] and and the proposed splitting algorithm, respectively. As aforementioned, model (8) is dividable: rows of *A *in the objective function and the constraints are independent. Hence, it is equivalent to *n *sub-problems:

(10) $$\begin{array}{c} \min_{a_{i}\in {\mathbb{R}}^{n}}\;\;{∥X_{1}^{T}a_{i}-X_{2,i}∥}_{2}^{2}+\gamma \langle \mu /\gamma w_{i}+\;1,\;\left|a_{i}\right|\rangle \\ \;\text{s.t.}\;\;\;\;\;∥a_{i}∥{\;}_{1}\leq 1 \end{array}$$

for *i *= 1, ..., *n*, where $X_{2,i}^{T}$ is the *i*-th row of the matrix $X_{2},\;1={\left[1,\;\ldots ,\;1\right]}^{T}\in {\mathbb{R}}^{n},w_{i}^{T},a_{i}^{T}$ is the *i*-th row of *W *and *A*, respectively. Sub-problem (10) can be transformed into a standard (convex) quadratic programming, and solved by software packages such as Mosek or CVX [18].

### The Block PCA model

The Block PCA model is motivated by Robust PCA model [19]

(11) $$\begin{array}{c} \min_{L,E}\;\;\;\;{∥L∥}_{*}+\lambda {∥E∥}_{1}\\ \;\text{s}.\text{t}.\;\;\;\;D=L+E, \end{array}$$

which ||*L*||_* _is the nuclear norm of the matrix *L*, and *D *is a given matrix.

The block PCA aims to seek a block submatrix in *D *by solving optimization problem

(12) $$\begin{array}{c} \min_{L,E}\;\;{∥L∥}_{*}+\lambda_{1}\langle W1,\;|L|\rangle +\lambda_{2}{∥E∥}_{1}\\ \;\text{s}.\text{t}.\;\;\;\;D=L+E \end{array}$$

where *W*1 is a weight matrix with all elements nonnegative.

In Block PCA, *D *∈ ℝ*^n, n ^*is set to be matrix $1_{n,n}\in {\mathbb{R}}^{n\times n}$ with all 1's, λ_2 _is constantly set as $1/\;\sqrt{n},$ and λ_1_∈(0, λ_2_). For a network with *n *nodes, we define weight matrix *W*1 = (*w*1_*ij*_)_*n × n*_, where

(13) $$w1_{i,j}={\left(p_{ij}/\;p_{0}\right)}^{2},$$

*p_ij _*is the length of the shortest path between the node *i *and *j, p*_0 _≥ 0 is a parameter less than the diameter of the network.

As in Robust PCA (11), the nuclear norm || · ||_* _usually induces a low rank matrix and the *l*_1 _norm || · ||_1 _induces a sparse matrix [19,20]. The constraint *D *= *L *+ *E *enforces to split matrix *D *into a low rank matrix *L *and a sparse matrix *E*. Different with Robust PCA, the Block PCA adds an extra term *λ*_1 _〈*W*1, |*L*|〉 = *λ*_1 _∑*w*1_*ij*_·|*L_ij_*| to (11). The nonnegative weight matrix *W*1 stands for the prior knowledge about low rank matrix *L*.

### Splitting algorithm for solving Block PCA

Block PCA model (12) can be transformed to a linear semidefinite programming (SDP)

(14) $$\begin{array}{c} \underset{{}_{W_{1},W_{2},L,E}}{\min}\;\;\frac{1}{2}\left[\text{trace}\left(W_{1}\right)+\text{trace}\left(W_{2}\right)\right]+\lambda_{1}\langle W1,|L|\rangle \\ \;\;\;\;\;\;\;\;+\lambda_{2}\langle 1_{n,n},\;|E|\rangle \\ \;\text{s}.\text{t}.\;\;\;\;\left[\begin{array}{c} W_{1}\;L\\ L^{T}\;W_{2} \end{array}\right]\underline{≻}\;0;\\ \;\;\;\;\;L+E=D. \end{array}$$

However, this transformation increases the size of the variable matrix from *n × n *to 2*n × *2*n*. Existing SDP solvers such as CVX [18] can not solve large-scale SDP problems. Instead, we solve Block PCA problem (12) by extending the splitting method [21] for optimization problem

(15) $$\begin{array}{c} \min\;\sum_{i=1}^{m}\theta_{i}\left(x_{i}\right)\\ \text{s}\text{.t}\text{.}\;\;\sum_{i=1}^{m}A_{i}x_{i}=b. \end{array}$$

Where *θ_i_*: ${\mathbb{R}}^{n_{i}}\to \mathbb{R}$ are closed convex functions, $A_{i}\in {\mathbb{R}}^{l\times n_{i}}$, *b*∈ℝ*^l^*.

Note that Block PCA (12) can be recast as

(16) $$\begin{array}{c} \underset{L,E,U}{\min}\;\;\;{∥L∥}_{*}+\lambda_{1}\langle W1,\;|U|\rangle +\lambda_{2}{∥E∥}_{1}\\ \text{s}.\text{t}.\;\;\left[\begin{array}{c} D\\ 0 \end{array}\right]=\left[\begin{array}{c} L\\ L \end{array}\right]+\left[\begin{array}{c} E\\ 0 \end{array}\right]-\left[\begin{array}{c} 0\\ U \end{array}\right]. \end{array}$$

By letting *θ*_1_(·): = ||·||_*_, *θ*_2_(·): = *λ*_1_〈*W*1, |*·*|〉, *θ*_3_(·): = *λ*_2_||·||_1_, and $b=\left[\begin{array}{c} D\\ 0 \end{array}\right],A_{1}L=\left[\begin{array}{c} L\\ L \end{array}\right],A_{2}U=-\left[\begin{array}{c} 0\\ U \end{array}\right]$ and $A_{3}E=\left[\begin{array}{c} E\\ 0 \end{array}\right],$ Block PCA (12) can be treated as a generalized case of (15) with matrix variables *L, E, U *and linear operators $A_{1},\;A_{2},\;\;A_{3}.$

Under the framework of [21], we next present an implementable splitting algorithm for the Block PCA model (12).

Define operator

(17) $$S_{\varepsilon }\left[t\right]=\left\{\begin{array}{cc} t-\varepsilon , & \text{if}\;t>\varepsilon ,\\ t+\varepsilon , & \;\;\;\text{if}\;t<-\varepsilon ,\\ 0, & \;\;\;\text{otherwise,} \end{array}\right)$$

*t *∈ ℝ and *ε *> 0. It can be extended to an arbitrary matrix *X *∈ ℝ^*n, n *^by applying element-wise operation, denoted by $S_{\varepsilon }\left[X\right]$.

Consider the sigular value decompostion (SVD) of the matrix *X*

(18) $$X=U\Sigma V^{T},$$

where *U *and *V *are orthogonal matrices consisting of singular vectors, and Σ is the diagnal matrix made up of the singular values. For each τ *>*0, the *soft-thresholding operator *$D_{\tau }$ is defined as [22]

(19) $$D_{\tau }\left(X\right)=U^{}S_{\tau }\left(\Sigma \right)V^{T}.$$

More generally, for a matrix *W *∈ ℝ*^n, n ^*with all elements nonnegative, we define

(20) $$S_{W}\left[X\right]=\left({\hat{x}}_{ij}\right),\;\;{\hat{x}}_{ij}=S_{w_{ij}}\left[x_{ij}\right].$$

Particularly, if *W *is the matrix with all elements 1, ||*X*||_*w *_degenerates to ||X||_1_, and $S_{\varepsilon W}\left[X\right]$ degenerates to $S_{\varepsilon }\left[X\right]$.

Let $\beta >0,\mu >2,\Lambda^{k}=\left[\begin{array}{c} \begin{array}{c} \Lambda_{1}^{k}\\ \Lambda_{2}^{k} \end{array} \end{array}\right],$ where $\Lambda_{1}^{k},\Lambda_{2}^{k}\in {\mathbb{R}}^{n,n}$. Then, for the calculated (*L^k^, E^k^, U*^k^, Λ^*k*^), the steps for each iterative (*L*^*k*+1^, *E*^*k*+1^, *U*^*k*+1^,Λ^*k*+1^) for solving (12) are as follows.

**Step 1**. Solve *L*^*k*+1 ^by the following problem.

(21) $$\begin{array}{c} \min_{L}\;\;\;||L|{|}_{*}-\langle \Lambda_{1}^{k}+\Lambda_{2}^{k},L\rangle +\frac{\beta }{2}||L+E^{k}-D|{|}^{2}\\ \;+\frac{\beta }{2}||L-U^{k}|{|}^{2} \end{array}$$

By Theorem 2.1 in [22], the unique solution of (21) is

(22) $$L^{k+1}=D_{\tau }\left[Y\right],$$

where $\tau =\frac{1}{2\beta },Y=\frac{1}{2}\left[D-E^{k}+U^{k}\right]+\frac{1}{2\beta }\left[\Lambda_{1}^{k}+\Lambda_{2}^{k}\right].$

**Step 2**. Update the Lagrangian multiplier $\Lambda^{k+\frac{1}{2}}\;\text{by}\;L^{k+1}.$

(23) $$\left\{\begin{array}{ccc} \Lambda_{1}^{k+\frac{1}{2}} & \;= & \;\Lambda_{1}^{k}-\beta \left(L^{k+1}+E^{k}-D\right),\\ \Lambda_{2}^{k+\frac{1}{2}} & \;= & \;\Lambda_{2}^{k}-\beta \left(L^{k+1}-U^{k}\right). \end{array}\right)$$

**Step 3**. Solve *U*^*k*+1^, *E*^*k*+1 ^by the following two problem.

$$\begin{array}{c} \underset{U}{\min}\left\{{∥U∥}_{\lambda_{1}W_{1}}+\langle \Lambda_{2}^{k+\frac{1}{2}},\;U\rangle +\frac{\beta \mu }{2}{∥U-U^{k}∥}^{2}\right\},\\ \underset{E}{\min}\left\{{∥E∥}_{1}-\langle \frac{1}{\lambda_{2}}\Lambda_{1}^{k+\frac{1}{2}},\;E\rangle +\frac{\beta \mu }{2\lambda_{2}}{∥E-E^{k}∥}^{2}\right\}. \end{array}$$

By the property of the operator *S_τ _*[*Y *] shown in [23],

(24) $$U^{k+1}=S_{\tau \lambda_{1}W_{1}}\left[Ũ\right],$$

where $\tau =\frac{1}{\beta \mu },Ũ=U^{k}-\frac{1}{\beta \mu }\Lambda_{2}^{k+\frac{1}{2}},$

(25) $$E^{k+1}=S_{\alpha }\left[Ẽ\right],$$

where $\alpha =\frac{\lambda_{2}}{\beta \mu },Ẽ=E^{k}+\frac{1}{\beta \mu }\Lambda_{1}^{k+\frac{1}{2}}.$

**Step 4**. Update the Lagrange multiplier Λ^*k*+1 ^by *L*^*k*+1^, *E*^*k*+1^.

(26) $$\begin{array}{c} \Lambda_{1}^{k+1}\;\;=\;\;\Lambda_{1}^{k}-\beta \left(L^{k+1}+E^{k+1}-D\right),\\ \Lambda_{2}^{k+1}\;\;=\;\;\Lambda_{2}^{k}-\beta \left(L^{k+1}-U^{k+1}\right). \end{array}$$

The algorithm can be terminated when

(27) $$\frac{\sqrt{{∥D-L^{k}-E^{k}∥}^{2}+{∥L^{k}-U^{k}∥}^{2}}}{∥D∥}\leq \;\varepsilon_{1}$$

and

(28) $$\sqrt{{∥\Delta L^{k}∥}^{2}+{∥\Delta E^{k}∥}^{2}+{∥\Delta U^{k}∥}^{2}}\leq \;\varepsilon_{2},$$

for tolerance *ε*_1 _*>*0, *ε*_2 _*>*0, where Δ*L^k ^*= *L*^*k*+1^- *L^k^*, Δ*E^k ^*= *E*^*k*+1 ^- *E^k^*, Δ*U^k ^*= *U*^*k*+1^- *U^k^*.

The splitting algorithm for solving Block PCA model is summarized in Algorithm 2.

**Algorithm 2:** 

Algorithm 2: Splitting algorithm	
**Input: ***W*1.	
**Output:**	low rank matrix *L*.
**Step 0**.	Initiation. Set *k *= 0, *β *> 0, *μ *> 2, $D=1_{n,n}$. Set *ε*_1 _*>*0, *ε*_2 _*>*0, $\lambda_{2}=1/\sqrt{n}$, *λ*_1 _∈ (0, *λ*_2_), *L*^0 ^= *D, E*^0 ^= 0, *U*^0 ^= *D*.
**Step 1**.	Solve *L*^*k*+1 ^by Eq. (22).
**Step 2**.	Update the Lagrangian multiplier ${\Lambda^{k}}^{+\frac{1}{2}}$ by Eq. (23).
**Step 3**.	Solve *U*^*k*+1^, *E*^*k*+1 ^via Eq. (24) and (25).
**Step 4**.	Update the Lagrange multiplier Λ^*k*+1 ^via Eq. (26).
**Step 5**.	Terminate if the stop criteria (27) and (28) are satisfied; otherwise, *k*: = *k *+ 1, goto Step 1.

In Algorithm 2, arguments *β *and *μ *are currently set constant. Adaptive settings of these arguments may speed up the convergence. The discussion of this issue in a simple case can be referred to [24].

## Results and discussion

We examine the NCI method based on two synthetic gene regulatory networks with different sizes. The GRN in first test is a small-sized network consisting of 14 genes and 27 interactions. There exist two communities in this GRN. In the second test, the network consists of 50 genes and 100 interactions and the data come from the Artificial Gene Network database [25]. Since the gene network is synthetic, the corresponding matrix *A *in (5) is known beforehand. We solve the GRN by the NCI method and compare it with *A *to evaluate the performance of the algorithm. Moreover, we examine the performance of the proposed splitting algorithm in the third test.

The metric used in the performance examination was introduced in [16]. It compares the signs of the estimated matrix *A_e _*with *A*. The accuracy is defined as

(29) $$accuracy=\left(r_{11}+r_{22}+r_{33}\right)\;/n^{2},$$

where *r*_11_, *r*_22_, and *r*_33 _are the number of correctly identified positives, zeros and negatives, representing promotions, repressions, and no interaction, respectively.

The algorithm runs on a computer with Pentium (R) dual-core CPU E5200 2.50GHz, and RAM 2.0GB. The parameters of the algorithm are chosen as follows. In Test 1, *γ *in problem (8) is chosen from {0.05, 0.02, 0.008} to find possible GRNs and *γ_τ _*= 0.02. In Test 2, *γ *is chosen from {0.02, 0.005, 0.001} and *γ_τ _*= 0.005. In the first two tests, *μ *is chosen as 10*γ *for problem (8), *λ*_1 _as 0.2λ_2 _in the Block PCA model, and *p*_0 _as $\frac{1}{4}d$ for Eq. (9), where *d *is the diameter of the corresponding network. The algorithm terminates in 3 iterations.

### Test 1. A small gene network with 14 genes

**Figure 1:**
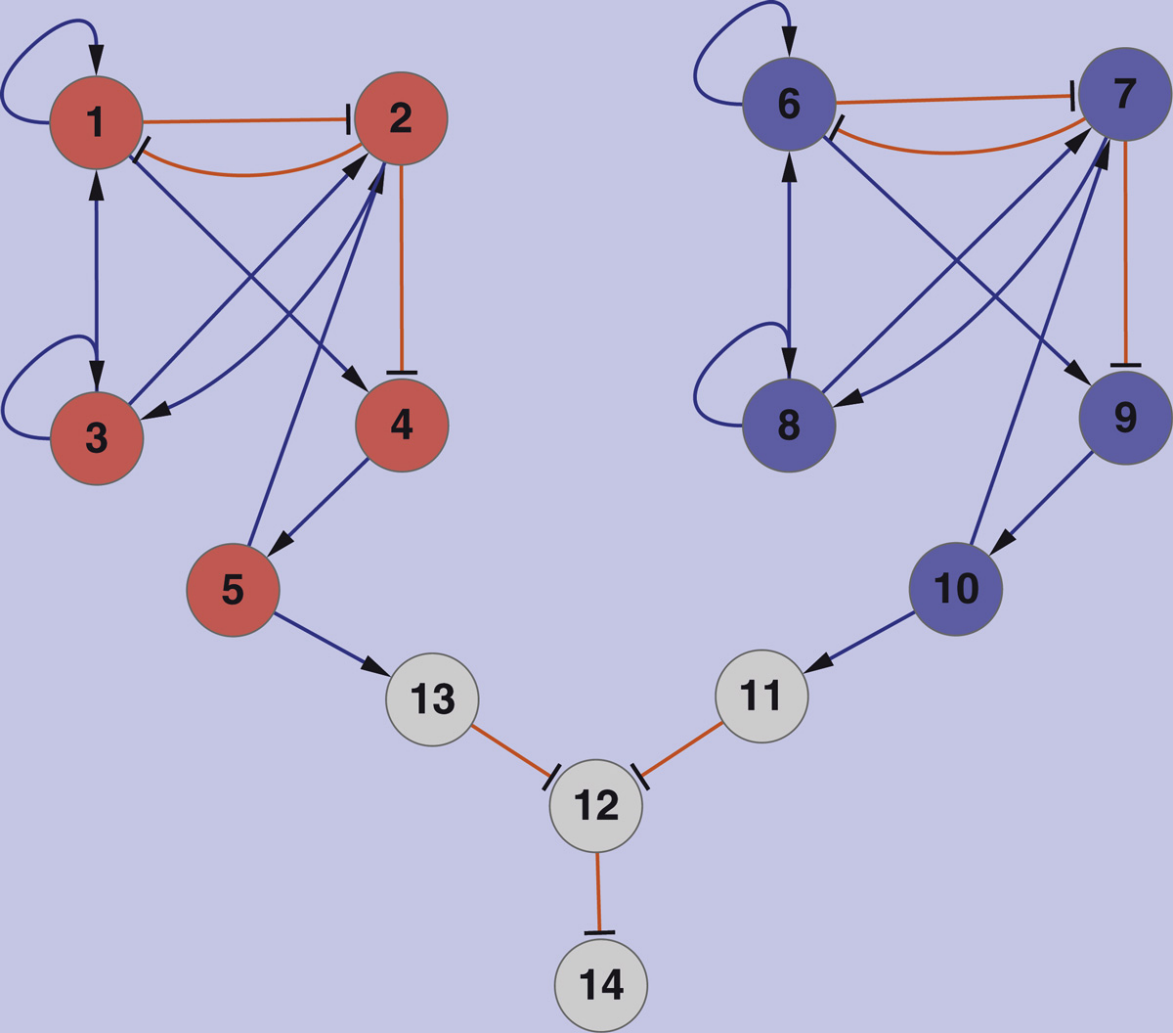
**Synthetic small gene network**. This synthetic gene network consists of 14 genes and 27 interactions. There are two communities: gene 1-5 form a community, gene 6-10 form the other. "→" indicates promotion interaction, while "⊣" indicates repression.

**Figure 2:**
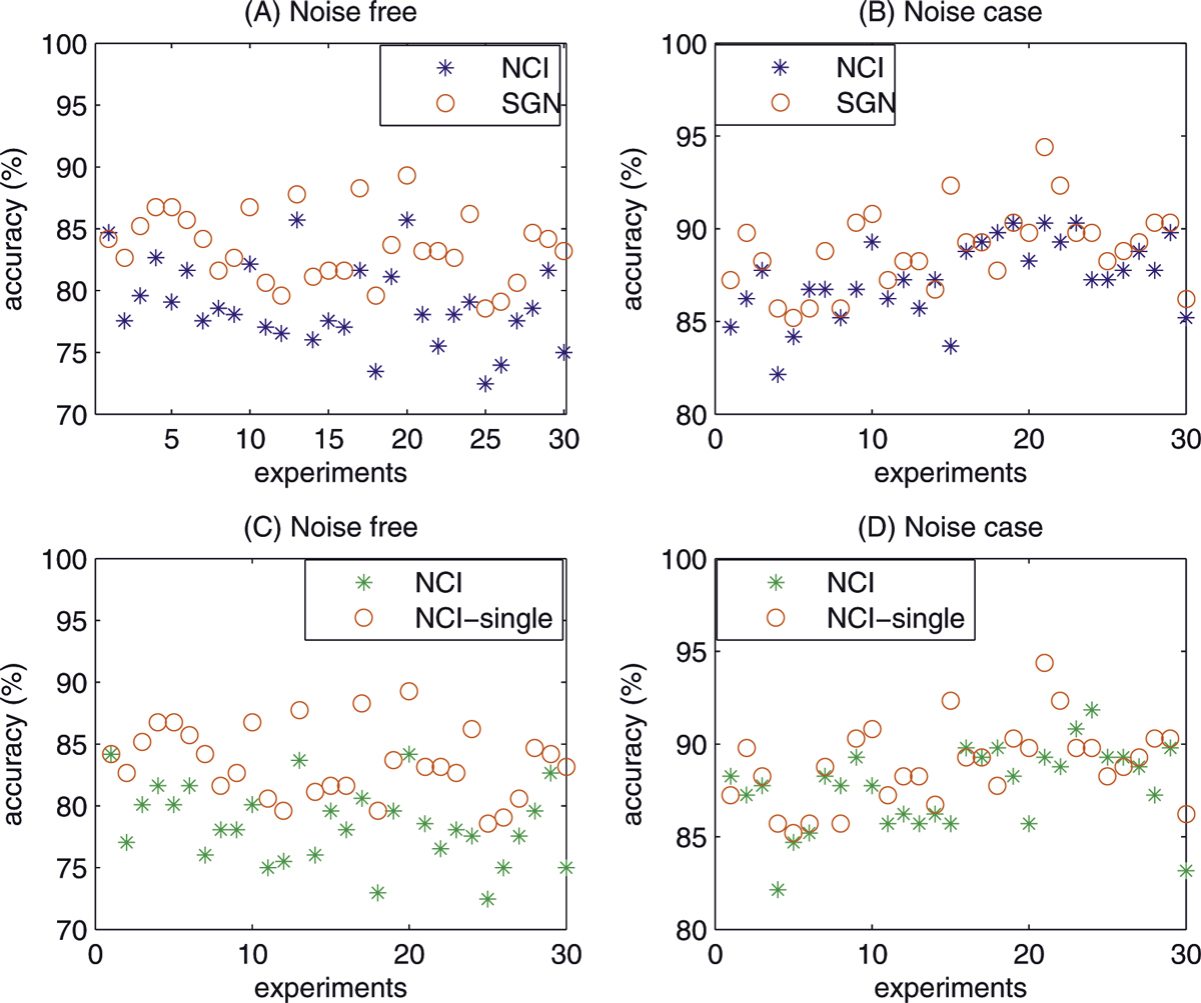
**Accuracy of NCI with the 30 runs**. The accuracies of the NCI method and SGN method are shown in (A), (B) with 30 random experiments. (C), (D) shows the efficiency of searching multiple possible GRNs at N-step of NCI method. "NCI" indicates normal NCI method searching multiple possible GRNs, "SGN" denotes the sparse gene regulatory network method [16], and "NCI-single" indicates the NCI method searching a single GRN at N-step. In (A) and (C), the expression levels are accurate, while in (B) and (D) 10% elements of the expression levels are incorporated with Gaussian noise with zero mean and unit variance.

Figure 1 shows the network and its two communities. The diameter of this gene network is 6. We choose different initial gene expression levels randomly for 30 times. The corresponding 30 accuracy rates of the calculated GRN are shown in Figure 2(A). "NCI" and "SGN" denote the NCI algorithm and sparse gene regulatory network method [16], respectively. Compared with the SGN algorithm, the NCI significantly improved prediction accuracy. In the noise case, 10% elements of the gene expression matrix *X *are incorporated with Gauss noise with zero mean and unit variance. The accuracy rates of two methods are shown in Figure 2(B). In both of the noise-free (Figure 2(A)) and noise cases (Figure 2(B)), the NCI method has much better performance in most of the 30 runs. In the noise-free case, the NCI algorithm increases the average accuracy from 78.8% to 83.5%. In the noise case, the NCI algorithm increases the average accuracy from 87.3% to 88.9%.

To show the effectiveness of the NCI method at N-step in searching multiple possible GRNs, we compare the accuracy rates with the results of one iteration at N-step (*γ *= *γ_τ _*at N-step). As shown in Figure 2(C), the average accuracy is improved from 78.5% to 83.5% in the 30 runs. In noise case (Figure 2(D)), the average accuracy is improved from 87.6% to 88.9%. Thus, a number of iterations at N-step is necessary for finding accurate GRNs with the NCI algorithm.

### Test 2. A gene network with 50 genes

**Figure 3:**
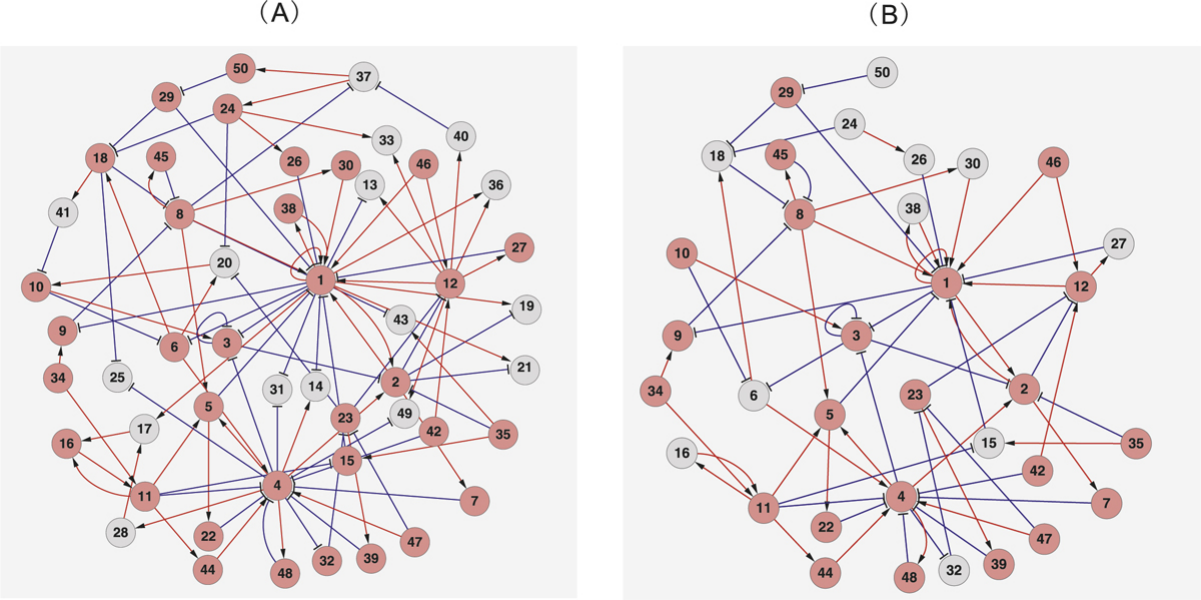
**Inference of the web100-023 gene regulatory network**. The web100-023 gene network consists of 50 genes and 100 interactions which is shown in (A), where "→" indicates promotion interaction, while "┤" indicates repression. The 34 nodes in red in (A) form a unique community. With the 34 genes in the true community, the community identified by the NCI algorithm has an overlap of 23 important genes (with large in-degree and out-degree). The overlap is indicated by nodes in red in (B).

The network in the second test consists of 50 genes and 100 interactions (See Figure 3(A)). Network statistics are listed in Table 1. The nodes in red in Figure 3(A)) form a unique community. The inferred network by NCI algorithm contains 41 genes and 87 interactions. As shown in Figure 3(B), the community identified by the NCI algorithm has a very large overlap with the true community. Among 34 genes in the true community, 23 important ones (with large in-degree and out-degree) are successfully identified.

**Table 1:** Characteristics of web100-023 network

number of vertices	50	number of arcs	100
density	0.04	in-degree center	Node 1
diameter	10	out-degree center	Node 1
characteristic path length	13.0612	closeness center	Node 14
average clustering coefficient	0.0747	betweeness center	Node 1

These statistics come from [27].

### Test 3. The performance of the Block PCA and splitting algorithm

**Figure 4:**
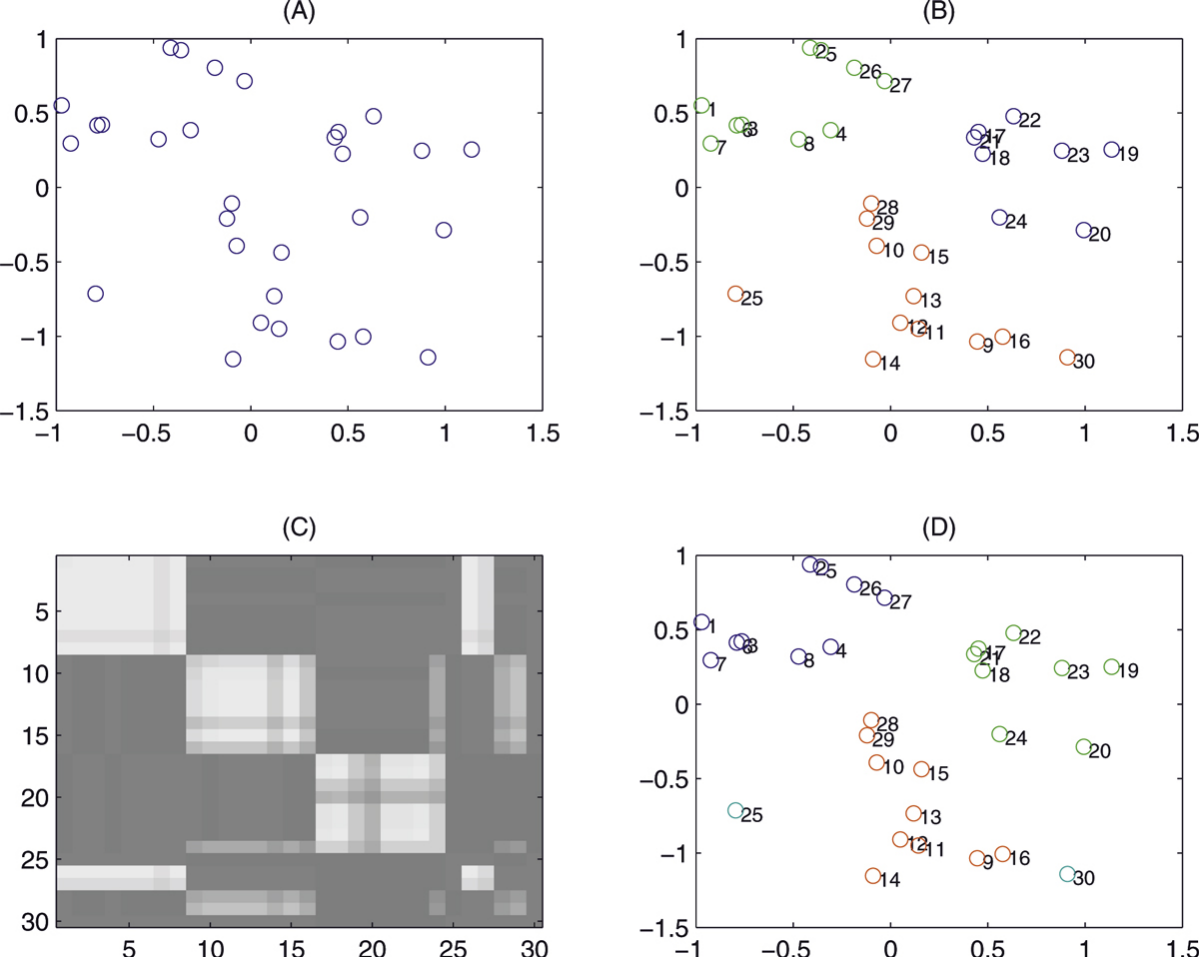
**Clusters produced by Block PCA and K-means**. In (A), 30 points are randomly generated on a plane. Three clusters calculated by K-means are shown in (B). The low rank matrix calculated by the splitting algorithm is depicted in (C). The corresponding three clusters calculated by SSVD are displayed with different colors depicted in (D). Among 30 data points, two points (the point 25 and 30) are outliers, not included in any cluster. The clustering result of the remaining 28 points is identical with that of K-means.

The following experiments are specially designed to test the efficiency of the Block PCA method and the performance of the splitting algorithm as well. We randomly generate three clusters with 30 points (See Figure 4(A)). Three clusters calculated by K-means are shown in Figure 4(B). Based on the distances between these points, matrix *W*1 is calculated by Eq. (13) with *p*_0 _= 0.684. The results of the splitting algorithm are shown in Figure 4(C). The corresponding three clusters calculated by SSVD are displayed with different colors in Figure 4(D). Among 30 data points, two points (the point 25 and 30) are outliers, not included in any cluster. The clustering result of the remaining 28 points is identical with that of K-means.

**Figure 5:**
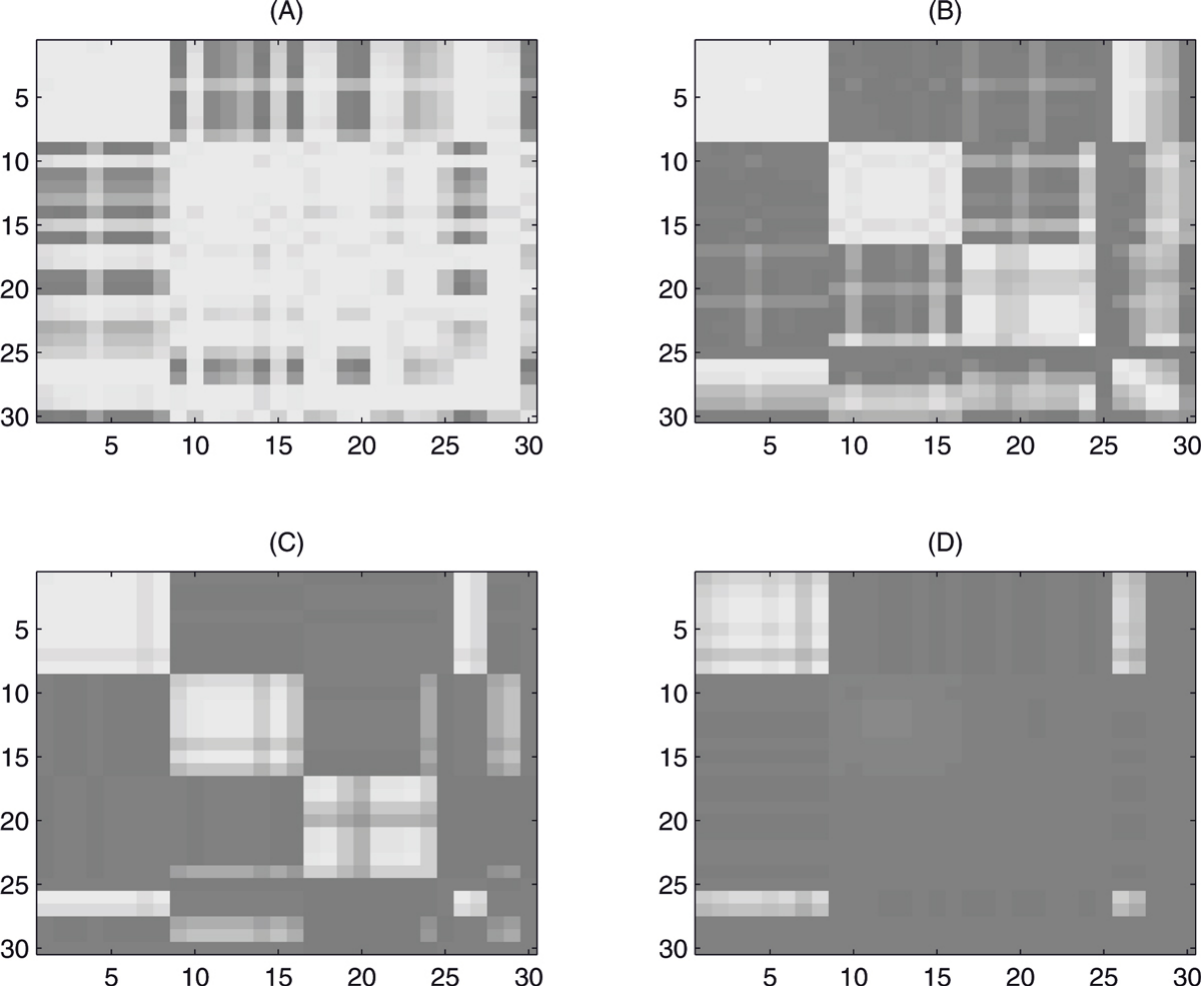
**Low rank matrices of the Block PCA model with various *λ*_1_**. The figure depicts the low rank matrices *L *of the Block PCA model with different values of *λ*_1_. In (A), (B), (C) and (D), the value of λ_1 _of the Block PCA model is chosen as 0.2*λ*_2_, 0.4*λ*_2_, 0.6*λ*_2_, and 0.7*λ*_2_, respectively.

To verify the effectiveness of the argument *λ*_1_, we choose different values and the calculated low rank matrices *L *are shown in Figure 5. It is shown that the number of nonzero elements of *L *(white pixels of the images) decreases, as *λ*_1 _increases. The numbers of nonzero elements of *L *are 804, 485, 284 and 100, with *λ*_1 _= 0.2*λ*_2_, 0.4*λ*_2_, 0.6*λ*_2_, and 0.7*λ*_2 _in Figure 5(A), (B), (C) and 5(D), respectively.

We compare the performance of the splitting algorithm with CVX and SDPNAL [26] by which the Block PCA model is solved via the SDP formulation (14). The results are listed in Table 2, where "Points30" indicates calculating on the data of 30 points on a plane, "funVal" indicates the calculated objective function value for the Block PCA model, "split", "cvx" and "sdpnal" indicate splitting method, CVX and SDPNAL, respectively. It is shown in Table 2 that splitting algorithm outperforms others in all the tests.

**Table 2:** Results of splitting algorithm, CVX and SDPNAL for solving the Block PCA model

		Points30	Points50	Points100	Points200	Points500
funVal	split	146.4654	301.4004	793.5010	2.1104e3	8.0980e3
	cvx	146.4654	--	--	--	--
	sdpnal	149.9589	307.0276	794.4018	2.1192e3	8.1217e3

||*D - L - E*||	split	3.0041e-5	1.0667e-5	7.5166e-5	1.4488e-4	3.7525e-4
	cvx	1.2590e-6	--	--	--	--
	sdpnal	4.2797e-5	2.3411e-4	9.1722e-4	0.0033	0.0057

||*L*||_*_	split	29.5328	54.8451	118.4007	260.8353	720.8204
	cvx	29.5323	--	--	--	--
	sdpnal	29.8736	55.2079	119.9717	261.0082	707.0530

〈*W*, |*L*|〉	split	631.1912	1.5986e3	7.0579e3	2.7437e4	1.8056e5
	cvx	631.1811	--	--	--	--
	sdpnal	700.7054	1.7995e3	7.0395e3	2.8083e4	1.8329e5

||*E*||_1_	split	451.1092	1.2638e3	4.6336e3	1.7925e4	1.1079e5
	cvx	451.1147	--	--	--	--
	sdpnal	447.5222	1.2408e3	4.6324e3	1.7854e4	1.1081e5

elapsed time(s)	split	4.88	19.66	80.43	687.65	1.4342e4
	cvx	6.74	--	--	--	--
	sdpnal	78.99	265.48	1.3231e3	6.9678e3	5.1222e4

"Points30" indicates the data with 30 points on a plane, and similarly for "Points50", ..., "Points500". "funVal" represents the calculated objective function value of the Block PCA model. "--" indicates out of memory. "2.1104e3" indicates 2.1104 × 10^3^. The implementation of SDPNAL is version 0 [28] and CVX is version 1.21 [29]. The SDPT3 engine is chosen for the CVX solver. The two arguments for the splitting algorithm are set as follows: *μ *is constantly set 5 for all tests, *β *is set 20/*n *for Points30, 200/*n *for Points50, Points100, Points200, and 500/*n *for Points500, where *n *is the number of points.

## Conclusion

We have developed the NCI method for gene regulatory network reconstruction from gene expression data. Based on the convex programming technology, the NCI method has shown the capability to identify multiple subnetworks within a large-scale gene regulatory network. The NCI method includes two main steps. At the first step, the algorithm infers a gene regulatory network. At the second step, the algorithm estimates potential community structures. These two steps repeat until the algorithm terminates. Furthermore, we have proposed an efficient Block PCA method for exploring communities within a GRN and the splitting algorithm for the Block PCA model. Numerical experiments have validated the effectiveness of the NCI method in identifying GRNs and inferring the communities.

## Abbreviations

GRN: gene regulatory network; NCI: network and community identification; Block PCA: block principal component analysis.

## Competing interests

The authors declare that they have no competing interests.

## Authors' contributions

XL and ZX designed the NCI method and wrote the manuscript. XL and LZ designed the Block PCA method and splitting algorithm. XL and FW designed the ODE GRN model and experiments. All authors read and approved the final manuscript.
